# Editorial: Brain plasticity following sensory loss: from basic mechanisms to therapy

**DOI:** 10.3389/fnins.2023.1331086

**Published:** 2023-11-17

**Authors:** Ron Kupers, Maurice Ptito

**Affiliations:** ^1^Department of Neuroscience, Panum Institute, University of Copenhagen, Copenhagen, Denmark; ^2^École d'optométrie, Université de Montréal, Montreal, QC, Canada

**Keywords:** vision, audition, brain plasticity, brain imaging, somethesis, cortex, rehabilitation

There is now ample evidence that both congenital blindness (CB) and acquired or late-onset blindness (LB) trigger a myriad of brain neuroplastic changes. With the advent of modern non-invasive brain imaging techniques such as positron emission tomography (PET), (functional) magnetic resonance imaging (f)MRI, magnetoencephalography (MEG) and diffusion imaging, the scientific study of the mechanisms mediating brain plasticity following sensory loss has gathered momentum over the past decades. The first brain imaging studies focused largely on blindness and revealed that the visually deprived occipital cortex at rest is metabolically hyperactive. Ensuing studies showed that tactile input (e.g., Braille reading) and other forms of non-visual input activate the occipital cortex in CB and to a lesser extent also in LB subjects. Brain morphometric and diffusion imaging studies have further shed light on the associated brain structural changes. Although most of the initial work strongly focused on the absence of vision, later studies also dealt with brain plastic changes following loss of auditory input, and to a lesser extent following loss of smell, taste, and somatosensory input. With this Research Topic, we wanted to further our understanding of the mechanisms underlying brain plasticity following sensory loss, to highlight similarities and differences between sensory loss in different sensory domains (e.g., vision, audition and somesthesis), and to identify major challenges for novel avenues for therapeutic progress. Finally, studies of the sensory-deprived brain also help us to shed new light on normal brain development and function.

Therefore, this Research Topic of Frontiers in Neuroscience is timely and brings a collection of ground-breaking novel research on the effects of sensory loss on neuroplastic processes in humans and in animal models. We present nine original articles and two systematic reviews that will contribute to expand our knowledge of brain reorganization following sensory loss.

## Description of the contents

The majority of the papers in this Research Topic deal with alterations in the visual system. Arend et al. report that blind individuals have changes in cortical gyrification, an anatomical measure that has not been previously reported in this context. The authors show an increase in gyrification in several brain areas of CB individuals and, importantly, a negative correlation between gyrification and cortical thickness in several different cortical areas. The authors discuss the impact of their results in relation to brain development and plasticity. Yizhar et al. used fMRI to study the role of the extrastriate body area (EBA) in action-related functions in CB individuals. Their findings indicate that the absence of visual experience does not favorize the development of action-related responses in the EBA. Moreover, CB participants showed a decrease in functional connectivity of the EBA with sensorimotor cortices, whereas connectivity with perception-related visual occipital cortices remained high. The authors further demonstrated that action-related functions and connectivity of the visual cortex are dependent on visuomotor experience. Bleau et al. present a meta-analysis of the neural substrates of spatial processing and navigation in blind individuals through touch and audition. The meta-analysis reveals that most studies agree that CB individuals recruit the same neural pathways as sighted controls when processing non-visual spatial information. The meta-analysis further shows that the primary visual cortex and associative occipital areas are involved in visuo-spatial processing via cross-modal plasticity mechanisms. The authors discuss the results in terms of the *amodality* hypothesis of spatial representations. Arbel et al. present novel data on face recognition in CB individuals. The authors trained a group of CB participants to use a visual-to-auditory sensory substitution device to recognize faces, whereafter they participated in an fMRI study. The results showed activation of the fusiform gyrus and other face-responsive-regions of the ventral visual stream. The authors concluded that there is a predisposition for sensory-independent and computation-specific processing in specific cortical regions that is independent of previous perceptual experience and that is pertained following sensory deprivation. Nadvar et al. studied resting state functional connectivity (rsFC) of area V1 following sight restoration in patients with retinitis pigmentosa who were implanted with the Argus II retinal prosthesis which partially restores vision. The aim was to test whether sight restoration with this treatment would reverse, in full or partly, the plastic changes induced by the vision loss. Their results showed that the decrease in rsFC between V1 and the post-central gyrus in CB participants was partially reversed by vision restoration. The authors suggest that rsFC between the occipital and somatosensory cortices could provide a biomarker for functional plastic changes following vision recovery. Maimon et al. report on visual perception in a small but unique group of children who had undergone vision-restoring cataract removal surgery as part of the Himalayan Cataract Project. Some of the children in the study were born with cataracts and gained a sense of sight for the first time, whereas others suffered late-onset blindness in one eye alone. The authors discuss their findings in the context of Molyneux's problem, i.e., the ability to correlate vision with touch quickly following sight restoration in blind individuals, and Hubel and Wiesel's theory of critical periods.

Two papers relate to plasticity following auditory deprivation, one in humans and a second one in animals. Grégoire et al. performed a meta-analysis of the literature on brain plastic changes following hearing loss at birth or later in life. Hearing loss is a growing problem in modern Western societies due to an aging population. Moreover, knowledge of brain neuroplastic changes could help to understand some disappointing results with cochlear implants, and therefore could improve hearing rehabilitation. The literature research revealed that the most consistent finding in deaf individuals was a volumetric decrease in gray matter around the auditory cortex. In deaf children, an additional volumetric decrease was reported in both gray and white matter at the level of the visual cortex. Grégoire et al. further discuss the role of confounding factors that could affect brain plasticity in deaf individuals such as the use of sign language and hearing aids, and frequently observed associated vestibular dysfunction or neurocognitive impairments. Using kittens rendered deaf, Mitzetlfelt et al. investigated the stimulus-driven neural activity associated with visual localization. The researchers recorded visual evoked potentials (VEPs) in response to visual stimuli presented at various eccentricities in the visual field. Their results showed no significant changes in VEPs in deaf cats that could explain the previously observed behavioral advantage. The authors concluded that cross-modal plasticity in deafness does not play a major role in cortical processing of the peripheral visual field.

Two studies report on patient groups with either central or peripheral lesions. Araneda et al. used diffusion MRI to study changes in white matter (WM) architecture in the geniculo-striate pathway in 40 children with unilateral spastic cerebral palsy (USCP). The authors report several alterations in diffusion imaging parameters of the optic radiations on the lesional compared with the non-lesional hemisphere. Both the nature and the side of the lesion (left or right hemisphere) had an impact on the type and magnitude of the WM changes. In USCP with periventricular and right-hemispheric lesions, the diffusion imaging parameters correlated with the patients' visuospatial assessment. Dedry et al. studied three unique patients with unilateral vocal fold paralysis. The patients were followed for 1 year with multiparametric voice assessments and longitudinal fMRI during a sustained phonation task and rsfMRI. One patient received an augmentation injection in the paralyzed vocal fold. This patient showed a bilateral activation of the voice-related nuclei in the brainstem during sustained phonation. In addition, rsFC between the voice motor/sensory brainstem nuclei and other voice-related ROIs correlated with mean airflow measures in this patient. This observation supports the hypothesis that promoting proprioceptive feedback, by temporarily rehabilitating glottic closure, can enhance the neural recovery process.

Finally, the study by Vaessen et al. addressed the question whether there is an abstract representation of emotions in the brain that is shared across stimulus types (face, body, voice) and sensory origin (visual, auditory). Thereto, the authors studied fMRI responses to ecological types of emotion expressions of different types and modalities. Using multivariate statistical analyses, the authors showed that there is a specific brain organization for affective signals which depends on stimulus category and modality. These findings are consistent with the notion that emotion expressions conveyed by different stimulus types have different functional roles in triggering rapid adaptive behavior.

We hope that the papers presented in this Research Topic of Frontiers in Neuroscience will contribute to a better understanding of the mechanisms of cross-modal plasticity following different forms of sensory loss and of sensory substitution and other restorative therapies that may lead to restoration of the lost functions.

## Author contributions

RK: Conceptualization, Validation, Writing—original draft, Writing—review & editing, Project administration. MP: Project administration, Validation, Writing—original draft, Writing—review & editing.

